# Marginal Impact of Brown Seaweed *Ascophyllum nodosum* and *Fucus vesiculosus* Extract on Metabolic and Inflammatory Response in Overweight and Obese Prediabetic Subjects

**DOI:** 10.3390/md20030174

**Published:** 2022-02-26

**Authors:** Marlène Vodouhè, Julie Marois, Valérie Guay, Nadine Leblanc, Stanley John Weisnagel, Jean-François Bilodeau, Hélène Jacques

**Affiliations:** 1 School of Nutrition, Faculty of Agricultural and Food Sciences, Université Laval, Québec City, QC G1V 0A6, Canada; marlene.vodouhe.1@ulaval.ca; 2 Institute of Nutrition and Functional Foods, Université Laval, Québec City, QC G1V 0A6, Canada; julie.marois@fsaa.ulaval.ca (J.M.); valerie.guay@fsaa.ulaval.ca (V.G.); nadine.leblanc@fsaa.ulaval.ca (N.L.); 3 Department of Medicine, Faculty of Medicine, Université Laval, CHU de Québec-Université Laval Research Centre, Québec City, QC G1V 4G2, Canada; john.weisnagel@crchudequebec.ulaval.ca (S.J.W.); jean-francois.bilodeau@crchudequebec.ulaval.ca (J.-F.B.)

**Keywords:** brown seaweed extract, metabolic and inflammatory markers, prediabetes

## Abstract

The objective of the present study was to test whether a brown seaweed extract rich in polyphenols combined with a low-calorie diet would induce additional weight loss and improve blood glucose homeostasis in association with a metabolic and inflammatory response in overweight/obese prediabetic subjects. Fifty-six overweight/obese, dysglycemic, and insulin-resistant men and women completed a randomized, placebo-controlled, double-blind, and parallel clinical trial. Subjects were administrated 500 mg/d of either brown seaweed extract or placebo combined with individualized nutritional advice for moderate weight loss over a period of 12 weeks. Glycemic, anthropometric, blood pressure, heart rate, body composition, lipid profile, gut integrity, and oxidative and inflammatory markers were measured before and at the end of the trial. No effect was observed on blood glucose. We observed significant but small decreases in plasma C-peptide at 120 min during 2 h-OGTT (3218 ± 181 at pre-intervention vs. 2865 ± 186 pmol/L at post-intervention in the brown seaweed group; 3004 ± 199 at pre-intervention vs. 2954 ± 179 pmol/L at post-intervention in the placebo group; changes between the two groups, *p* = 0.002), heart rate (72 ± 10 at pre-intervention vs. 69 ± 9 (*n*/min) at post-intervention in the brown seaweed group; 68 ± 9 at pre-intervention vs. 68 ± 8 (*n*/min) at post-intervention in the placebo group; changes between the two groups, *p* = 0.01), and an inhibition in the increase of pro-inflammatory interleukin-6 (IL-6) (1.3 ± 0.7 at pre-intervention vs. 1.5 ± 0.7 pg/L at post-intervention in the brown seaweed group; 1.4 ± 1.1 at pre-intervention vs. 2.2 ± 1.6 pg/L at post-intervention in the placebo group; changes between the two groups, *p* = 0.02) following brown seaweed consumption compared with placebo in the context of moderate weight loss. Although consumption of brown seaweed extract had no effect on body weight or blood glucose, an early attenuation of the inflammatory response was observed in association with marginal changes in metabolic parameters related to the prevention of diabetes type 2.

## 1. Introduction

The growing prevalence of type 2 diabetes worldwide is particularly alarming. Based on recent world statistics [1], 352 million adults aged from 20 to 79 years old were glucose intolerant (7.3%), and 425 million (8.8%) had type 2 diabetes in 2017 [1]. The association between the dramatic increase of the disease and the rising prevalence of obesity is often highlighted [2]. Obesity and overweight initiate several metabolic disorders [3] that include insulin resistance, which is considered to be the central feature leading to type 2 diabetes [4]. Prediabetes is an intermediate stage in the development of type 2 diabetes and is characterized by abnormal high fasting glucose, impaired glucose tolerance, and/or high-fasting insulin [5]. A number of other metabolic dysfunctions are linked to prediabetes and type 2 diabetes, such as chronic systemic low-grade inflammation [6,7], oxidative stress, endothelial dysfunction [8], and impaired gut integrity [9]. The extent to which these phenomena are observed differs with respect to diet composition, among other environmental factors. These disorders may be reversible at the prediabetes stage when nutritional interventions are implemented.

In a clinical setting, the implementation of a weight-loss approach is generally the first-line intervention in the prevention and management of type 2 diabetes among overweight or obese people. The main recommended nutritional treatment to lose weight and prevent type 2 diabetes is the reduction of energy intake [10]. Caloric restriction and weight loss have been shown to improve insulin sensitivity and reduce hyperglycemia [11,12]. A moderate caloric intake restriction of approximately 2093 KJ (500 kcal) per day is recommended [10,13] for a weight loss of 2 to 4 kg/month as suggested by the 2006 Canadian Obesity Guidelines [14].

Marine organisms are rich sources of compounds with diverse biological activities [15]. Among the most promising bioactive molecules in preventing type 2 diabetes are the phlorotannins, a class of polyphenols exclusively produced by brown seaweeds [16]. In vitro and in vivo studies [17,18] show that phlorotannin-rich extracts of brown seaweed *Ascophyllum nodosum* and *Fucus vesiculosus* reduce the α-amylase and α-glucosidase activities involved in the digestion of carbohydrates, resulting in a 90% reduction of glucose 30 min after a meal and a 40% decrease in peak insulin secretion in mice, as reported by Roy et al. [17]. In humans, it has been demonstrated that a single acute dose of 500 mg of a polyphenol-rich extract of *Ascophyllum nodosum* and *Fucus vesiculosus* had no significant effect on glycemia but induced a 12% reduction in the insulin incremental area under the curve (IAUC) following consumption of 50 g of bread compared with a placebo in healthy adults [19]. Fucoidan, a polysaccharide found in brown seaweeds, has also been recognized as a promising bioactive molecule with glucose-lowering potential [20,21], via a reduction of α-amylase and α-glucosidase activities, and therefore a diminution of intestinal absorption of glucose, and an enhancement of cellular glucose uptake via insulin through modulation of cellular glucose transporter GLUT-4 and AMP-activated protein kinase (AMPK). However, there are few studies addressing the chronic physiological effects of brown seaweed extract on metabolic and inflammatory markers in humans and particularly in people at high risk of type 2 diabetes. 

Achieving normal blood glucose levels is one of the major goals in diabetes prevention, and it can be reached by different means, such as weight loss or an improvement of glucose homeostasis through a nutritional supplement [22]. From a biological perspective, the response to an energy-restricted diet varies between individuals [23], and this strategy alone is somewhat less effective in the long term [24]. Therefore, a nutritional supplement is often necessary in adjunction to energy restriction [25]. In this context, a brown seaweed extract, which has been shown to reduce waist circumference [26], could be administered in addition to a reduced-calorie diet to obtain additional effects. 

The present study was thus undertaken to evaluate the effect of daily consumption of 500 mg of brown seaweed (*Ascophyllum nodosum* and *Fucus vesiculosus*) capsules rich in polyphenols associated with a reduced-energy diet on body weight and blood glucose homeostasis (glucose, insulin, and C-peptide in the fasting state and during a 2 h oral glucose tolerance test) for 12 weeks in overweight prediabetic subjects. Lipid profile, heart rate, markers of inflammation, oxidative stress, and integrity of the intestinal barrier were measured as secondary outcomes. We expect that brown seaweed extract lowers body weight and improves glucose homeostasis and metabolic and inflammatory markers in the context of moderate weight loss in this 12-week clinical trial.

## 2. Results

### 2.1. Demographic, Baseline Characteristics, and Compliance 

In total, 56 subjects were enrolled in the study with 61% (*n* = 34) women and 39% (*n* = 22) men. Participants were comparable for age, body weight, BMI, waist and hip circumferences, systolic and diastolic blood pressures, heart rate, lipid profile, fasting glycemia, and OGTT 2 h glycemia (Supplementary Table S1). Forty-four subjects had impaired fasting glucose (5.6–6.9 mmol/L), 22 had high blood glucose levels after a 2 h test (7.7–11.0 mmol/L), and 24 had elevated glycated hemoglobin concentration (5.5–6.4%). Among those, 31 participants met one criterion, whereas 16 and 9 participants met two and three criteria, respectively. 

### 2.2. Food Intake and Physical Activity 

Food Frequency Questionnaire data show no difference in energy and macronutrient intake between groups at the baseline and after the 12-week intervention. The mean reduction in daily energy intake of the brown seaweed group was similar to that of the placebo group (*p* = 0.90). With regards to physical activity, no difference within and between groups was observed (Supplementary Table S2). 

### 2.3. Anthropometric Measurement, Body Composition, Blood Pressure, and Heart Rate

**Figure 1:**
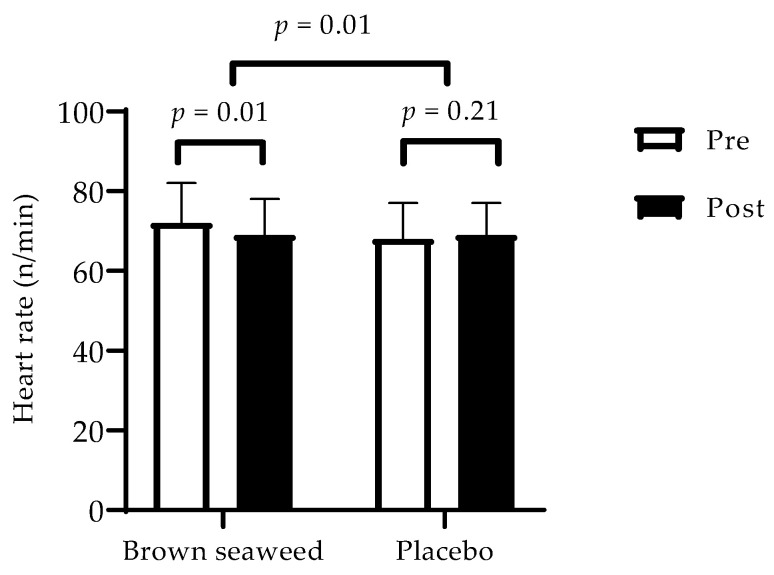
Variations of heart rate over time within and between groups. □ Pre-intervention (at the beginning of the 12-week trial). ▪ Post-intervention (at the end of the 12-week trial). A repeated-measures ANOVA with two factors (treatment, post-pre phase) was performed to compare changes over time (post-pre) between groups. Values are means with standard deviations represented by vertical bars. *n* = 56.

There was no difference in anthropometric, body composition, and blood pressure parameters between the groups (Table 1). However, intragroup analysis revealed reductions in body weight, BMI, waist circumference, and total fat mass from the baseline in the two groups (*p* ≤ 0.01) (Table 1). Results showed a 4% decrease in heart rate in the brown seaweed group and a 1.5% decrease in the placebo group. When we compared changes from the baseline (post vs. pre) between the two treatments, brown seaweed extract showed a reduction in the heart rate compared with control (*p* = 0.01) (Figure 1).

### 2.4. Fasting Glycemic, Lipid, and Hepatic Biomarkers in the Fasting State 

There were no significant differences in glycemic, lipid, and hepatic parameters in the fasting state between the two groups (Table 2). 

### 2.5. Glucose, Insulin, and C-Peptide during OGTT 

**Figure 2:**
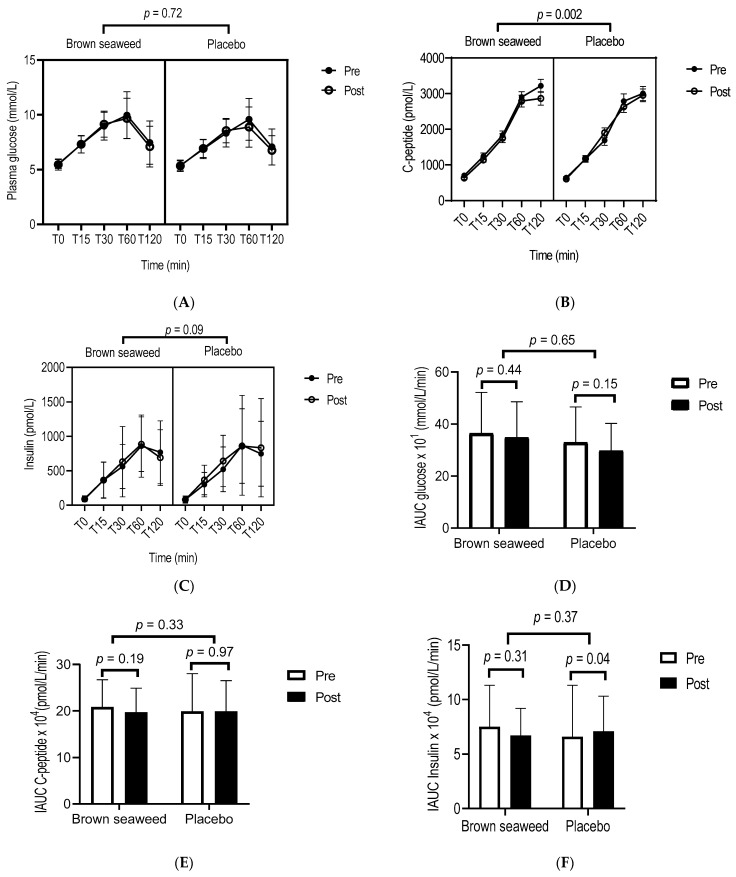
Glucose, insulin and C-peptide concentrations and IAUC during OGTT. □ Pre-intervention (at the beginning of the 12-week trial). ■ Post-intervention (at the end of the 12-week trial). Repeated measures ANOVA highlighting significant differences in the concentrations of glucose (**A**), C-peptide (**B**), and insulin (**C**), and in their respective IAUCs (**D**–**F**) over time between the brown seaweed and the placebo groups. A mixed model for repeated-measures ANOVA with three factors (treatment, OGTT time points, post-pre phase) (**A**–**C**) and with two factors (treatment, post-pre phase) (**D**–**F**) was performed. Incremental area under the curve (IAUC) for glucose, insulin, and C-peptide during the 2 h-OGTT was calculated from the trapezoidal rule and fasting baseline values. Values are means with standard deviations represented by vertical bars. *n* = 56 for glucose, *n* = 44 for insulin, and *n* = 56 for C-peptide.

We performed repeated-measures ANOVA for glucose (Figure 2A), C-peptide (Figure 2B), and insulin (Figure 2C) up to 120 min during OGTT. A reduction was observed in C-peptide concentrations at 120 min during OGTT in the brown seaweed group compared with the placebo group (*p* = 0.002) (Figure 2B). There were no differences between the two groups for fasting glucose, insulin, and C-peptide (Table 2) and their corresponding mean IAUC up to 120 min after OGTT (Figure 2D–F). 

### 2.6. Inflammatory Status, Oxidative Stress Status, and Gut Integrity

**Figure 3:**
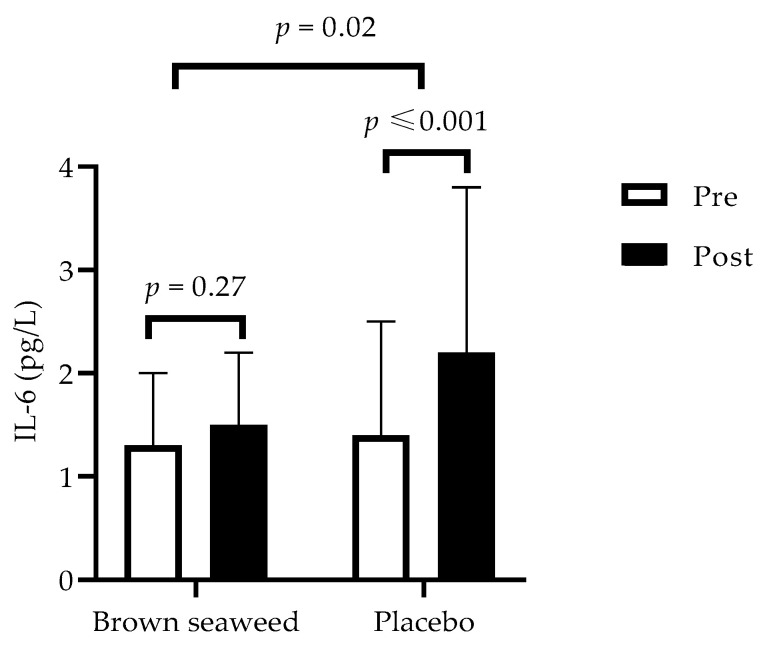
Variations of IL-6 concentrations over time within and between groups. □ Pre-intervention (at the beginning of the 12-week trial). ■ Post-intervention (at the end of the trial). A repeated-measures ANOVA with two factors (treatment, post-pre phase) was performed to compare changes over time (post-pre) between groups. Values are means with standard deviations represented by vertical bars. *n* = 56.

High-sensitivity C-reactive protein (hsCRP) and F_2_-isoprostane (8-iso-PGF_2α_) responses were similar between treatments (Table 3). However, there was a lower increase of plasma interleukin-6 (IL-6) concentration in the brown seaweed group (15%) compared with the placebo group (57%) (*p* = 0.02) (Figure 3). There was no difference in the gut integrity parameters between the groups. 

### 2.7. Adverse Effects 

No serious adverse event was reported in this study.

## 3. Discussion

The study investigated the effect of brown seaweed (*Ascophyllum nodosum* and *Fucus vesiculosus*) extract (InSea2^®^) rich in polyphenols combined with a moderate weight loss on glycemic and metabolic control among prediabetic overweight/obese subjects over 12 months. In this study, we advised both groups to follow a moderate energy-restricted balanced diet. Therefore, body weight, waist circumferences, and total fat mass decreased significantly in both groups. The main findings in this study indicate that daily consumption of 500 mg of brown seaweed extract had no effect on body weight and blood glucose, our primary outcomes, but had a modest beneficial impact on insulin secretion through the reduction of late C-peptide secretion during 2 h OGTT, heart rate and on the inhibition of the rise of IL-6 concentration in plasma compared with placebo in the context of moderate weight loss. 

### 3.1. Weight Loss 

With respect to anthropometric parameters, our results showed that the improvement in body weight and composition induced by consumption of 500 mg per day brown seaweed extract rich in polyphenols with dietary modifications aimed at moderate weight loss was not greater than that provoked by dietary modifications alone. The lack of additional weight loss is consistent with recent data from Derosa et al. [27], who reported no effect of 6-month administration of food supplement containing *Ascophyllum nodosum* and *Fucus vesiculosus* extract and chromium picolinate on body weight and BMI in dysglycemic subjects. On the other hand, De Martin and co-authors [26] reported a significant decrease in waist circumference in overweight and obese subjects after 6 months of treatment with the same nutraceutical combination that had been administered in the study by Derosa et al. [27]. In light of these studies, it is possible that a reducing effect of brown seaweed extract on body weight was masked by the effect of the low-calorie diet in the present study. It is also to be considered that the absence of additional weight loss could be attributed to the dysglycemic status of our subjects or to the absence of chromium picolinate in the supplement.

### 3.2. Glucose, Insulin, and C-Peptide 

The lack of effect on blood glucose could be due, on the one hand, to the lack of effect on body weight [28] and, on the other hand, to taking the supplement once a day rather than three times a day (before breakfast, lunch, and dinner), as performed De Martin et al. [26]. There is a possibility that a single administration of the supplement intake during the day might have resulted in a less pronounced effect on the activities of intestinal α-amylase and α-glucosidase and, therefore, on the absorption of the glucose and blood levels.

It should also be considered that blood glucose is maintained primarily because of regulated pancreatic insulin secretion. In this respect, C-peptide is a byproduct of insulin synthesis from pro-insulin. It is secreted by pancreatic β-cells in equal amounts with insulin; however, unlike insulin, C-peptide is not extracted by the liver and has a constant peripheral clearance. It is considered a valuable and precise biomarker of insulin secretion due to its longer time life in circulation (20 to 30 min for C-peptide versus 5 min for insulin) [29]. Moreover, C-peptide hypersecretion occurred in prediabetic and diabetic patients in the fasting state and in the early and late phases of the OGTT [30]. In this context, a reduction in plasma C-peptide in these patients is desirable. In this study, we observed a modest decrease of C-peptide at the late phase of OGTT in participants consuming brown seaweed extract, suggesting a potential for reduced insulin secretion in the long term, thereby decreasing the risk for developing glucose intolerance and type 2 diabetes [31,32]. 

This effect may be associated with phlorotannins, polyphenolic compounds found in brown seaweeds only, and fucoidan, found as a sulfated polysaccharide in brown marine algae, known to decrease the α-amylase and α-glucosidase activities and, therefore, the digestion and assimilation of glucose [17,18,20,33]. This may result, in the long term, in an improvement of pancreatic function and, ultimately, in a reduction of glucose intolerance seen in prediabetic patients. Our results are in good agreement, but less pronounced than those of De Martin et al. [26] and Derosa et al. [27], who have recently reported a decrease in glycemia, insulin secretion, insulin resistance [26], and postprandial glycemia [27] following a 12-week consumption of 712.5 mg of *Ascophyllum nodosum* and *Fucus vesiculosus* extract with the addition of chromium picolinate. In light of these studies [26,27], it is important to consider that the presence of picolinate chromium, known as a hypoglycemic agent, could have accentuated the impact of the *Ascophyllum nodosum* and *Fucus vesiculosus* extract on glycemic response. Furthermore, in both previous studies [26,27], the effects were more pronounced after 6 months than after 3 months of treatment with an additional reduction of fasting plasma glucose, glycated hemoglobin, and insulin resistance. The differences in the impact of the extract on glucose homeostasis observed in the present study and the two former studies [26,27] could be explained by the frequency and doses, duration of the trial, and use of picolinate of chromium in the previous studies. However, our study is one of few to have explored the effects of brown seaweed extract alone and show that there is a beneficial impact, albeit modest, of the brown seaweed extract per se, without chromium picolinate, on insulin secretion.

### 3.3. Lipid Profile, Inflammation, and Heart Rate 

Lipid-associated risk for cardiovascular disease events is gradual and continuous. LDL carries cholesterol from the liver throughout the body and leaves excess on the endothelium of the arteries, causing atherosclerotic plaques to develop. Consequently, a surplus of LDL cholesterol is associated with an increased risk of cardiovascular events in patients both with and without type 2 diabetes [34]. Therefore, as a primary goal of therapy, target LDL cholesterol levels for adults with diabetes are <2.60 mmol/L [35]. In the present study, no effect was observed on lipid parameters. Our results are not in accordance with those of Shin et al. [36], who observed a decrease in total and LDL cholesterol following daily consumption of 144 mg of *Ecklonia cava* brown seaweed during 12 weeks among hypercholesterolemic subjects, nor with those of Iacoviello et al. [37] who reported a decrease in triglycerides after the consumption of a high dose (900 mg) of *Ascophyllum nodosum* extract over 6 weeks. The difference could be explained by the nature of the seaweed, the dose, and the duration of the trial. Further studies with higher doses of *Ascophyllum nodosum* and *Fucus vesiculosus* extract are suggested to assess the impact of its consumption on lipid profile among people at risk of type 2 diabetes.

Inflammation and oxidative stress may play a pivotal role in the pathophysiology of type 2 diabetes. The elevated inflammatory and oxidative biomarkers observed at the post-intervention stage could be explained by the chronic inflammation process that is implicated in diabetes. Among several inflammatory biomarkers, Il-6 has been shown to predict the development of type 2 diabetes, promoting the development of inflammation, insulin resistance, and β-cell dysfunction [38]. In the present study, our results indicate that the extract inhibited the increase in IL-6 observed in overweight/obese placebo subjects, thereby attenuating early pro-inflammatory response, and suggesting that consumption of the extract might have potential anti-inflammatory properties. In line with this result, it has already been shown that *Ascophyllum nodosum* extract can inhibit pro-inflammatory IL-6 gene expression in the porcine colon ex vivo and in vitro [39,40]. Phlorotannins are proposed as the main compounds in marine brown seaweeds associated with this effect. Indeed, as reported by Catarino et al. [41], phlorotannins can inhibit the expression of pro-inflammatory cytokines and interfering with transcriptional regulation. Furthermore, in the present study, variations in Il-6 were strongly and positively correlated with glycemia in the fasting state (*r* = 50, *p* = 0.001). Furthermore, in post-intervention, we observed trends between Il-6 and insulin resistance measured by HOMA-IR (*r* = 26, *p* = 0.07), and Il-6 and plasma insulin values at 120 min during OGTT (*r* = 0.24, *p* = 0.10). These results suggest that the inhibition of IL-6 increase could be a mechanism responsible for the observed effects of the brown seaweed extract on parameters related to glucose homeostasis. However, in the present study, hs-CRP was not affected by treatment; therefore, indicating no effect of brown seaweed extract on the pro-inflammatory late regulation process. Interestingly, a late anti-inflammatory response, characterized by a decrease in inflammatory biomarkers TNF-α and hsCRP, was observed in an earlier 6-month study [27] investigating the metabolic and inflammatory effects of an extract of *Ascophyllum nodosum* and *Fucus vesiculosus* combined with chromium picolinate, therefore supporting the concept that the addition of chromium to brown seaweed extract might modulate inflammation differently [42].

Interestingly, heart rate is associated with inflammation [43] and plasma IL-6 concentrations [44]. We indeed observed a positive correlation between variations in heart rate and Il-6 (*r* = 0.29, *p* = 0.04). Therefore, the reduced heart rate seen in the brown seaweed group could be linked to the inhibition of increased pro-inflammatory marker Il-6. Interestingly, Jung et al. [45] previously observed that dieckol, a phlorotannin present in brown seaweed, can induce in vitro inhibition of cyclooxygenase 2 expression and prostaglandin E(2) production. 

### 3.4. Integrity of Gut Barrier 

Dysregulation of intestinal mucosal barrier function and loss of integrity of the gut barrier can increase the passage to the intestinal pathogens and endotoxins, which cause infection or inflammation [46]. Animal models have provided evidence linking gut barrier dysfunction, activation of inflammation signaling pathways, and progression of insulin resistance to type 2 diabetes [47]. The potential of *Ecklonia radiata*, a brown seaweed from New Zealand, and its polysaccharides (fucoidan and alginate) were reported to improve gut health in a rat model by increasing stool bulk, short-chain fatty acids, and butyrate production [48]. However, Michiels et al. [49] showed no effect of a dried extract of *Ascophyllum nodosum* on gut microbiota in piglets. To our best knowledge, no studies have addressed the impact of brown seaweed on the health and integrity of the gut barrier in humans. In the context of our study, no effect of our brown seaweed extract was observed on LBP, an acute-phase protein secreted by the liver in the bloodstream in response to a bacterial infection, and zonulin, a protein associated with a loss of intestinal barrier function that modulates the permeability of tight junctions between cells of the wall of the digestive tract. Further studies measuring various parameters of gut integrity, such as occludin, claudins, or mucin, in addition to LBP and zonulin, are necessary to determine the impact of brown seaweed and their extracts on gut integrity among people at risk of type 2 diabetes. 

The results of the present study showed a marginal beneficial impact of a polyphenol-rich extract obtained from two algae, *Ascophyllum nodosum* and *Fucus vesiculosus*, in the context of moderate weight loss over 12 weeks on metabolic risk factors of type 2 diabetes and cardiovascular disease, namely glucose homeostasis, heart rate, and Il-6 in prediabetic subjects. Moreover, our results should be interpreted with caution, given certain limitations. On the one hand, diet and physical activity were not strictly controlled, but on the other hand, our experimental approach was closer to the real context. Furthermore, we used 2 h OGTT instead of 3 h OGTT, which could have minimized the real effect of brown seaweed extract on glycemia, insulin, and C-peptide during OGTT. Finally, it might be worthwhile in subsequent studies to extend the duration of the trial to 6 months.

We acknowledge that this discussion has been focused on phlorotannins and fucoidans due to their known bioactive properties, but the extract contains other compounds such as small saccharides (mono- and disaccharide) as well as oligo- and polysaccharides, fatty acids, and phenolic compounds, as measured by Gabbia et al. [18]. For instance, the polysaccharide-rich composition of brown algae has shown the potential to act as prebiotics and to positively modulate the gut microbiota [50]. Moreover, in addition to phlorotannins, there are other polyphenolic compounds such as phenolic acids, flavonoids, phenolic terpenoids, and bromophenols in brown seaweeds, as reported by Cotas et al. [51]. On their own, each of these compounds can exhibit significant biological properties, including antidiabetic, anti-inflammatory, and antioxidant activities [51], and could potentially affect the parameters measured in this study. However, compared to phlorotannins, less is known about those polyphenols due to a lack of their characterization, isolation, and specific bioactivity analysis. Furthermore, it is possible that when combined, these compounds have interactions with each other, depending on the type and structure of these compounds, thereby modulating their effects. 

In conclusion, the present results indicate that the consumption of a brown seaweed extract of *Ascophyllum nodosum* and *Fucus vesiculosus* for 12 weeks had no effect on body weight and blood glucose but induced a marginal beneficial impact on insulin secretion, heart rate, and Il-6 in overweight/obese prediabetic subjects. These results suggest that early attenuation of the inflammatory response by 500 mg of brown seaweed extract in the context of moderate weight loss could be associated with modest changes in metabolic parameters related to the prevention of type 2 diabetes. More studies with different doses and intervention durations are needed to determine the effects of this supplement on cardiovascular and type 2 diabetes risk factors.

## 4. Materials and Methods 

### 4.1. Study Design

This study was a parallel, double-blind, randomized controlled 12-week clinical trial conducted from summer 2017 to winter 2018 at the Institute of Nutrition and Functional Foods in Quebec City (QC, Canada). The study was in accordance with the Declaration of Helsinki and was approved by the Université Laval’s health-science research ethics committee. Participants were given a detailed written informed consent form to read and sign prior to their participation in the study. This trial was registered at clinicaltrials.gov as NCT03075943.

### 4.2. Population 

Volunteers were recruited on a voluntary basis from the Institute of Nutrition and Functional Foods contacts database, Laval University network, and Diabète Québec Association website. Inclusion criteria included age between 18–70 years, body mass index (BMI) ≥ 27 cm, waist circumference ≥ 94 cm for men and ≥80 cm for women, fasting insulin ≥ 60 pmol/L, fasting glycemia between 5.6–6.9 mmol/L or/and 2 h glucose in oral glucose tolerance test (OGTT) between 7.8–11.0 mmol/L or/and glycated hemoglobin A1C (HbA1C) between 5.7–6.4% for prediabetes diagnostics [52], and stable weight during the last 3 months. Exclusion criteria included type 2 diabetes, uncontrolled hypertension, thyroid disorders, renal and hepatic dysfunction, gastro-intestinal disorders, or other chronic or acute diseases. People who intake medications, food supplements, or natural health products that affect glucose and lipid metabolisms or body weight were also excluded. Major surgery during the last 3 months, smoking, pregnancy or breastfeeding, and allergies to fish, seafood, or iodine were also considered as exclusion criteria. A total of 260 subjects, recruited in the Québec City metropolitan area through media advertising, were screened to examine their eligibility to participate in this study (Supplementary Figure S1). During the first screening visit, two self-administered online questionnaires were completed by all subjects to collect information on medical history, lifestyle, economic and socio-demographic characteristics. Of 260 subjects, 125 subjects were excluded, and 11 decided not to participate. During the second screening visit, blood was collected from 124 subjects, and 58 subjects were also excluded from the study because they did not meet the plasma inclusion criteria. Thus, of 66 eligible volunteers after the two screening visits, 56 completed the study (Supplementary Figure S1) because ten subjects among the eligible volunteers no longer met inclusion criteria, dropped out, or refused to pursue the study for personal reasons during the study. During a 2-week run-in phase, the participants were asked to maintain their dietary routine and refrain from vigorous activity. After the 2-week run-in phase, the participants were randomly assigned to either the placebo group or the brown seaweed extract group, based on weight, BMI, and gender, and received individualized nutritional counseling for a moderate weight loss. 

### 4.3. Intervention 

In the brown seaweed extract group, each participant received 500 mg of polyphenol-rich brown seaweed *Ascophyllum nodosum* and *Fucus vesiculosus* extract (2 capsules sold as a standardized extract under the registered trademark InSea2^®^ of InnoVactiv Inc. (Rimouski, QC, Canada) to be taken daily 30 min before the main meal of the day for 12 weeks). The extract was prepared using an exclusive hot water extraction, followed by filtration and ultrafiltration processes and spray-drying. The extract used in the present study contained algae polyphenols (35%, as indicated in the certificate of analysis of the commercial product). The extraction process removed alginates and part of the salts, and the remaining constituents were mostly composed of algae polysaccharides, of which fucoidans were 11.5% fucose and minerals (iodine content = 67 mg/kg). The ability of InSea2^®^ to inhibit α-amylase was 97.8% at a concentration of 0.1 g/L algae extract, as indicated in the certificate of analysis of the commercial product. Heavy metal analysis showed that inorganic arsenic (<0.41 mg/kg), cadmium (0.31 mg/kg), lead (0.77 mg/kg), and mercury (0.03 mg/kg) were less than the limit of quantification. The chemical characterization of InSea2^®^ has previously been described in detail [18], confirming that the extract is mainly composed of saccharide derivatives and contains ~35% algae polyphenols, of which phloroglucinol derivatives, measured by NMR analysis and revealing the presence of fatty acids by GC-MS.

Participants of the control group received the same dose of placebo capsule to be taken in the same conditions as described above. Treatment and placebo capsules were similar in form, color, and taste and were supplied by innoVactiv Cie (Rimouski, Canada). The treatment capsule contained extract of brown seaweed (250 mg), dibasic calcium phosphate (150 mg), microcrystalline cellulose (100 mg), croscarmellose (3 mg), magnesium stearate (5 mg), titanium dioxide (1.8 mg), and hypromellose (94.3 mg). Placebo capsule contained microcrystalline cellulose (191 mg), dibasic calcium phosphate (287 mg), magnesium stearate (5 mg), caramel (25 mg), titanium dioxide (1.8 mg), hypromellose (94.3 mg). 

The nutritional intervention aimed for moderate calorie restriction of about 500 kcal based on basal metabolism corrected for physical activity, using the traditional Harris Benedict–formula [53]. The individualized counseling intervention was given in the form of a dietary plan based on the “Food Guide for Diabetic Patients” [54] and “Health Plate Approach” [55]. Protein powders, fish oil, omega-3 or any marine supplements, as well as natural health products known to have effects on glucose or insulin levels, weight, satiety, or appetite, were forbidden. Moreover, it was not allowed to use weight loss products available on the market or to follow another weight loss program. In addition, slimming or anti-cellulite cosmetic creams were not permitted. A validated online Food Frequency Questionnaire [56] was completed by participants. Short International Physical Activity Questionnaire [57,58] and Side Effect Reporting Form were completed. Compliance was assessed by capsule counts [59]. Any change in medication, temporary medication, natural health product intake, or consumption of any other food supplements were monitored according to the exclusion criteria during the entire study period. To document compliance, subjects were asked to return unused capsules at the end of the study. Capsule counts indicated a minimum of 80% compliance in both groups.

### 4.4. Anthropometric and Body Composition Measurements 

Bodyweight, height, and waist circumference were measured using conventional measurements. BMI was calculated as weight (kg)/height^2^ (m^2^). Body composition (total mass, fat mass, visceral mass) was determined using a dual-energy X-ray absorptiometry (DEXA) scanner (GE Lunar Prodigy Bone Densitometer, GE Lunar Healthcare, Madison, WI, USA), and data were analyzed using Lunar Prodigy Advance-enCORE 2010 software version 14.1. 

### 4.5. Blood Pressure and Heart Rate Measurements 

Blood pressure and heart rate were measured three times on the right arm using an automatic tensiometer (Digital Blood Pressure Monitor, model HEM-907XL; OMRON^®^, Kyoto, Japan) following a 10 min rest at the beginning and at the end of the experimental period using a Digital Blood Pressure Monitor (model HEM-907XL; OMRON^®^ Blood pressure Monitor, Kyoto, Japan). 

### 4.6. Oral Glucose Tolerance Test (OGTT) 

At the beginning and end of the experimental periods, glucose tolerance was estimated by a two-hour, 75 g OGTT using a standard solution of glucose (Glucodex). Blood was collected before at T0 (0 min), T1 (15 min), T2 (30 min), T3 (60 min), and T4 (120 min). T0 (0 min) represented the fasting state.

### 4.7. Blood Collection and Storage 

Blood samples were collected following a 12 h overnight fasting state before each OGTT. Venous blood samples were taken in tubes containing ethylenediaminetetraacetic acid (EDTA) and immediately centrifuged at 1100× *g* for 10 min. Plasma was stored at −80 °C for further analysis of glycemic and lipid variables, inflammatory (hsCRP, IL-6), antioxidant (F_2_-isoprostane as 8-iso PGF_2α_ isomer), and gut integrity markers (LBP and zonulin).

### 4.8. Glycemia, Insulin, C-Peptide, and Glycated Hemoglobin A1C Measurements 

Glucose, insulin, and C-peptide concentrations were measured in fasting plasma and at different time points of the OGTT. Blood glucose was measured using The Dimension Vista^®^ GLU method (REF K1039) (Siemens Healthcare Diagnostics Inc., Tarrytown, NY, USA), an adaptation of the hexokinase-glucose-6-phosphate dehydrogenase method [60]. Insulin and C-peptide, a marker of insulin secretion, were determined using ADVIA Centaur XPT Immunoassay System test (Siemens Healthcare Diagnostics Inc., Tarrytown, NY, USA), a sandwich immunoassay using direct chemiluminescent technology, and specific insulin and C-peptide antibodies. HbA1c was determined using a 2.0 assay on a VARIANT II TURBO Hemoglobin Testing System (Bio-Rad Laboratories Inc., Hercules, CA, USA) using the principle of ion-exchange high-performance liquid chromatography. These analyses were performed at the Research Centre of Laval University Hospital (CHU of Québec) (QC, Canada). 

The Homeostasis Model Assessment of Insulin Resistance (HOMA-IR) was used to estimate insulin sensitivity/resistance. HOMA-IR index was calculated according to the following formula: fasting insulin concentration (µL/mL) × fasting glucose concentration (mmol/L)/22.5 [61]. 

### 4.9. Lipid Profile Biomarkers Measurements 

Plasma total triglycerides (TG), Low-Density Lipoprotein cholesterol (LDLc), High-Density Lipoprotein cholesterol (HDLc), Total cholesterol (Total chol), well-known markers of cardiovascular risk, were measured using enzymatic, colorimetric assay on a Cobas c701/702 instrument (Roche Diagnostics GmbH, Mannheim, Germany). Analyzes were performed at CHU of Québec (QC, Canada).

### 4.10. Hepatic Enzymes Measurements 

Aspartate aminotransferase (AST) and alanine aminotransferase (ALT), plasma markers of hepatic function, were measured by Dimension Vista^®^ AST and ALT methods, adapted from the recommended methods of International Federation of Clinical Chemistry [62] with the use of the coenzyme pyridoxal-5-phosphate (P5P). Laboratory analyzes were performed at CHU of Québec (QC, Canada).

### 4.11. Inflammatory, Oxidative Stress and Gut Integrity Biomarkers 

Hs-CRP was determined at CHU of Québec (QC, Canada) using immunonephelometric technique (Siemens BN Prospec, Siemens Healthineers, Oakville, ON, Canada). Interleukin-6 was measured in plasma using Multiplex kits (EMD Millipore, Etobicoke, ON, Canada), and plates were read using Bio-Plex 200 system (Bio-Rad, Mississauga, ON, Canada). Total F2-isoprostane (as 8-iso-PGF_2α_ isomer) level from non-enzymatic peroxidation of arachidonic acid, a biomarker of oxidative stress, was measured in plasma, as previously described [63]. The latter analyses were conducted at the Québec Heart and Lung Institute (QC, Canada). LBP and zonulin were determined in plasma using commercially available enzyme-linked immunosorbent assay kits from Biomatik (Cambridge, ON, Canada). The analyses were conducted at the Institute of Nutrition and Functional Foods (QC, Canada).

### 4.12. Statistical Analysis 

The sample size was determined based on insulin reduction after a single acute intake of 2 capsules of 250 mg of a polyphenol-enriched brown seaweed extract from data published by Paradis et al. [19] with a statistical power of 80%, yielding a sample size of 27 for each group. Statistical analyses were performed using the statistical analysis software SPSS (version 22, 2007, IBM SPSS Statistics), except for data from OGTT, which were analyzed using SAS 9.4 (SAS Institute, Cary, NC, USA. Results are presented as means ± standard deviation. The primary analysis was to compare plasma glucose, insulin, and C-peptide obtained with OGTT before and after the intervention between the placebo and treatment. For that purpose, a mixed model for repeated-measures ANOVA with three factors (treatment, OGTT time points, post-pre phase) was performed. Incremental area under the curve (IAUC) for glucose, insulin, and C-peptide during the 2 h-OGTT was calculated from the trapezoidal rule and fasting baseline values. A repeated-measures ANOVA test with two factors (treatment, post-pre phase) was performed to compare changes over time (post-pre) between groups for the other parameters. Prior to performing repeated-measures ANOVA, normality was checked using a Shapiro–Wilk test, and distribution was normalized using logarithmic transformation when needed. Student’s *t*-test (when data were normally distributed) and Mann–Whitney U test (when data were abnormally distributed) were performed to compare means at pre-intervention between groups, and F2-isoprostane changes from baseline between and within groups. Considering the small size of the experimental groups and the multiplicity of analysis, a Bonferroni correction was performed for the anthropometric (*p* ≤ 0.01), blood pressure and heart rate (*p* ≤ 0.02), glycemic (*p* ≤ 0.005), lipid (*p* ≤ 0.01), hepatic (*p* ≤ 0.02), inflammatory and oxidative stress (*p* ≤ 0.02), and gut integrity (*p* ≤ 0.03) markers. To detect associations between variables, Pearson correlation coefficients were calculated using the Pearson correlation (CORR) procedure. Differences were considered significant at *p* ≤ 0.05 (two-tailed) level.

## Figures and Tables

**Table 1:** Anthropometric, body composition, and blood pressure parameters over time within and between groups.

	Brown Seaweed Extract (*n* = 27)			Placebo (*n* = 29)			*p*_IxG_^3^
	Pre ^1^	Post ^1^	*p*_I_^2^	Pre ^1^	Post ^1^	*p*_I_^2^	
Weight (kg)	91 ± 13	89 ± 13	<0.001	91 ± 14	89 ± 14	0.003	0.51
BMI (kg/m^2^)	33 ± 4	32 ± 4	<0.001	33 ± 5	32 ± 4	0.003	0.55
Waist (cm)	109 ± 10	108 ± 10	0.003	108 ± 10	107 ± 10	0.007	0.76
Total fat mass (kg) †	38 ± 9	37 ± 9	0.001	39 ± 10	38 ± 10	0.001	0.93
Total lean mass (kg)	51 ± 8	50 ± 8	0.03	50 ± 10	50 ± 10	0.65	0.20
Visceral fat mass (kg)	1.9 ± 0.6	1.7 ± 0.6	0.04	1.6 ± 0.6	1.6 ± 0.5	0.23	0.52
SBP (mmHg)	119 ± 11	121 ± 12	0.27	118 ± 11	118 ± 12	0.93	0.39
DBP (mmHg)	75 ± 9	74 ± 9	0.51	76 ± 7	74 ± 8	0.14	0.58

BMI, body mass index; SBP, systolic blood pressure; DBP, diastolic blood pressure. † *n* = 56; *n* = 55 for total fat mass; *n* = 54 for visceral fat mass. ^1^ A repeated-measures ANOVA with two factors (treatment, post-pre phase) was performed to compare changes over time (post-pre) between groups. Mean ± SD pre-intervention (at the beginning of the 12-week trial) and post-intervention (at the end of the 12-week trial). ^2^ *p*-value to compare changes over time from the baseline to 12 weeks (pre vs. post) within each group (placebo or brown seaweed extract). ^3^ *p*-value to compare change over time (pre vs. post) between groups (placebo vs. brown seaweed extract).

**Table 2:** Glycemic, lipid, and hepatic markers over time within and between groups.

	Brown Seaweed Extract (*n* = 27)			Placebo (*n* = 29)			*p*_IxG_^3^
	Pre ^1^	Post ^1^	*p*_I_^2^	Pre ^1^	Post ^1^	*p*_I_^2^	
Fasting glucose (mmol/L)	5.8 ± 0.5	5.7 ± 0.4	0.14	5.7 ± 0.6	5.6 ± 0.5	0.45	0.60
Fasting insulin (pmol/L) †	129 ± 49	123 ± 48	0.22	123 ± 63	114 ± 73	0.49	0.76
Fasting C-peptide (pmol/L)	1009 ± 298	957 ± 284	0.06	945 ± 307	916 ± 306	0.26	0.60
Hemoglobin A1c (%)	5.6 ± 0.3	5.6 ± 0.3	0.83	5.6 ± 0.2	5.6 ± 0.2	0.46	0.72
Total chol (mmol/L)	5.5 ± 0.8	5.2 ± 0.8	0.07	5.1 ± 0.8	5.1 ± 0.7	0.48	0.07
Triglycerides (mmol/L)	1.5 ± 0.7	1.7 ± 0.8	0.20	1.5 ± 0.5	1.5 ± 0.4	0.55	0.60
LDLc (mmol/L)	3.1 ± 0.7	2.9 ± 0.8	0.06	3.0 ± 0.7	3.0 ± 0.6	0.36	0.05
HDLc (mmol/L)	1.4 ± 0.3	1.3 ± 0.2	0.001	1.3 ± 0.3	1.3 ± 0.3	0.25	0.09
Chol/HDLc	4.4 ± 0.8	4.4 ± 0.9	0.12	4.4 ± 0.9	4.4 ± 0.8	0.17	0.86
HOMA-IR	4.8 ± 1.9	4.5 ± 1.8	0.18	4.5 ± 2.5	4.0 ± 2.8	0.65	0.57
AST (U/L)	23 ± 8	20 ± 5	0.02	25 ± 11	20 ± 8	0.004	0.69
ALT (U/L)	24 ± 8	24 ± 8	0.99	28 ± 20	31 ± 18	0.04	0.15
AST/ALT	1.1 ± 0.5	0.9 ± 0.2	0.07	1.1 ± 0.4	0.8 ± 0.3	0.001	0.23

Total Chol, total cholesterol; LDLc, low-density lipoprotein cholesterol; HDLc, high-density lipoprotein cholesterol; HOMA-IR, homeostasic model assessment of insulin resistance; AST, aspartate aminotransferase; ALT, alanine aminotransferase. † *n* = 56; *n* = 53 for fasting insulin; *n* = 53 for HOMA-IR. ^1^ A repeated-measures ANOVA with two factors (treatment, post-pre phase) was performed to compare changes over time (post-pre) between groups. Mean ± SD pre-intervention (at beginning of 12-week trial) and post-intervention (at end of 12-week trial). ^2^ *p*-value to compare changes over time from the baseline to 12 weeks (pre vs. post) within each group (placebo or brown seaweed extract). ^3^ *p*-value to compare change over time (pre vs. post) between groups (placebo vs. brown seaweed extract).

**Table 3:** Inflammatory, oxidative stress, and gut barrier integrity markers over time within and between groups.

	Brown Seaweed Extract (*n* = 27)			Placebo (*n* = 29)			*p*_IxG_^3^
	Pre ^1^	Post ^1^	*p*_I_^2^	Pre ^1^	Post ^1^	*p*_I_^2^	
hsCRP (mg/L) †	3.0 ± 1.9	3.6 ± 2.7	0.67	2.7 ± 1.6	3.4 ± 2.6	0.88	0.84
F_2_-isoprostane (8-iso PGF_2__α_) × 10^−1^ ng/mL	2.2 ± 0.8	2.4 ± 0.8	0.65	2.5 ± 1.9	3.0 ± 2.3	0.53	0.76
LBP × 10^3^ (ng/mL)	6.4 ± 1.9	8.3 ± 1.8	0.002	6.8 ± 1.3	7.7 ± 1.9	0.17	0.20
Zonulin (ng/mL)	1.2 ± 0.6	1.2 ± 1.0	0.60	1.7 ± 1.0	1.9 ± 1.1	0.53	0.42

hsCRP, high-sensitivity C-reactive protein; LBP, lipopolysaccharide-binding protein. † *n* = 53 for hsCRP; *n* = 54 for LBP; *n* = 55 for zonulin. ^1^ A repeated-measures ANOVA with two factors (treatment, post-pre phase) was performed to compare changes over time (post-pre) between groups. Mean ± SD pre-intervention (at the beginning of the 12-week trial) and post-intervention (at the end of the 12-week trial). ^2^ *p*-value to compare changes over time from the baseline to 12 weeks (pre vs. post) within each group (placebo or brown seaweed extract). ^3^ *p*-value to compare change over time (pre vs. post) between groups (placebo vs. brown seaweed extract).

## Data Availability

Data available on request due to ethical restrictions.

## Supplementary Materials

The following are available online at https://www.mdpi.com/article/10.3390/md20030174/s1, Table S1: Subjects’ characteristics; Table S2: Nutritional intakes over time within and between groups; Figure S1: Flow chart.
