# Supplementary material for: Marginal Impact of Brown Seaweed Ascophyllum nodosum and Fucus vesiculosus Extract on Metabolic and Inflammatory Response in Overweight and Obese Prediabetic Subjects
Source: Mar Drugs. 2022 Feb 26;20(3):174. doi: 10.3390/md20030174 (PMC8951415; doi:10.3390/md20030174)
Supplement: Supplementary file 1 [file marinedrugs-20-00174-s001.zip › marinedrugs-1577658-supplementary.pdf]

*Article*

# Marginal Impact of Brown Seaweed *Ascophyllum nodosum* and *Fucus vesiculosus* eExtract on Metabolic and Inflammatory Response in Overweight and Obese Prediabetic Subjects

**Marlène Vodouhè [1], Julie Marois [2], Valérie Guay [2], Nadine Leblanc [2], Stanley John Weisnagel [3], Jean-François Bilodeau [3] and Héléne Jacques [1,*]**

**Supplemental Table S1. Subjects` characteristics**

| Variables | Brown seaweed extract ($n$ = 27) | Placebo ($n$ = 29) | P |
|---|---|---|---|
| Men/women (n/n) | 11/16 | 11/18 | - |
| Age (years) | 55±12 | 51±11 | 0.22 |
| Weight (kg) | 91±13 | 91±13 | 0.89 |
| BMI (Kg/m$^2$) | 33±4 | 33±4 | 0.89 |
| Waist circumference (cm) | 109±11 | 107±9 | 0.49 |
| Hip circumference (cm) | 113±2 | 115±2 | 0.38 |
| SBP (mmHg) | 126±14 | 122±13 | 0.32 |
| DBP (mmHg) | 76±10 | 76±8 | 0.90 |
| Heart beats (beats/min) | 74±10 | 70±9 | 0.13 |
| Total cholesterol (mmol/l) | 5.3±0.9 | 5.5±0.9 | 0.44 |
| TAG (mmol/l) | 1.6±0.8 | 1.6±0.6 | 0.92 |
| LDLc (mmol/l) | 3.1±0.8 | 3.3±0.8 | 0.40 |
| HDLc (mmol/l) | 1.4±0.3 | 1.4±0.4 | 0.97 |
| Chol/HDLc | 3.8±0.8 | 4.0±1 | 0.33 |
| Fasting glycemia (mmol/l) | 5.9±0.4 | 5.9±0.4 | 0.63 |
| 2h Glycemia (mmol/) | 7.2±1.6 | 7.4±1.5 | 0.60 |

BMI, Body Mass Index; SBP, Systolic Blood Pressure; DBP, Diastolic Blood Pressure; TAG, Total triglycerides; LDLc, Low Density Lipoprotein cholesterol; HDLc, High Density Lipoprotein cholesterol, Chol, Cholesterol.
Mean ± SD
*P* value to compare parameters between groups (placebo vs brown seaweed extract) at the screening, as performed by ANOVA.

**Supplemental Table S2. Nutritional intakes over time within and between groups**

| | Brown seaweed extract (n = 27) | | | Placebo (n = 29) | | | $P_{IXG}$[2,3] |
|---|---|---|---|---|---|---|---|
| | **Pre**[1] | **Post**[1] | $P_I$[2] | **Pre**[1] | **Post**[1] | $P_I$[2] | |
| Energy (KJ) | 9 971±737 | 8 870±737 | 0.08 | 10 666±712 | 9 059±720 | 0.05 | 0.90 |
| % Carbohydrate | 47±1 | 47±1 | 0.64 | 46±1 | 48±1 | 0.02 | 0.18 |
| % Protein | 17±1 | 18±1 | 0.01 | 17±1 | 18±1 | 0.22 | 0.24 |
| % Fat | 35±1 | 35±1 | 0.63 | 36±1 | 35±1 | 0.07 | 0.34 |
| Saturated fatty acid (g) | 32±3 | 27±3 | 0.08 | 35 ±3 | 28 ± 3 | 0.01 | 0.63 |
| MUFA (g) | 37±3 | 33±3 | 0.09 | 41±3 | 34±3 | 0.02 | 0.66 |
| PUFA (g) | 18±1 | 15±1 | 0.08 | 19±1 | 15±1 | 0.01 | 0.48 |
| Trans fatty acid (g) | 3.5±0.3 | 3.1±0.3 | 0.08 | 4.0±0.3 | 3.2±0.3 | 0.04 | 0.83 |
| Cholesterol (g) | 302±33 | 297±33 | 0.9 | 340±32 | 276±32 | 0.11 | 0.22 |
| Caffeine (g) | 235±30 | 224±30 | 0.51 | 172±29 | 175±29 | 0.83 | 0.53 |
| Alcohol (g) | 11±2 | 6±2 | 0.01 | 12±2 | 6±2 | 0.01 | 0.89 |
| Total fiber (g) | 27±2 | 27±2 | 0.79 | 29±2 | 30±2 | 0.36 | 0.66 |
| Insoluble fiber (g) | 18±1 | 18±1 | 0.78 | 19±1 | 20±1 | 0.30 | 0.59 |
| Physical activity (MET) | 2502±429 | 1970±429 | 0.25 | 1936±407 | 1622±414 | 0.86 | 0.48 |

MUFA, Monounsaturated Fatty Acid; PUFA, Polyunsaturated Fatty Acid.

1. Mean ± SD pre-intervention (at the beginning of the 12-week trial) and post-intervention (at the end of the 12-week trial)
2. *P value* to compare changes over time from the baseline to 12 weeks (Pre vs Post) within each group (placebo or brown seaweed extract), as performed by repeated-measures ANOVA test with two factors (treatment, post-pre phase).
3. *P value* interaction to compare change over time (Pre vs Post) between groups (placebo vs brown seaweed extract).

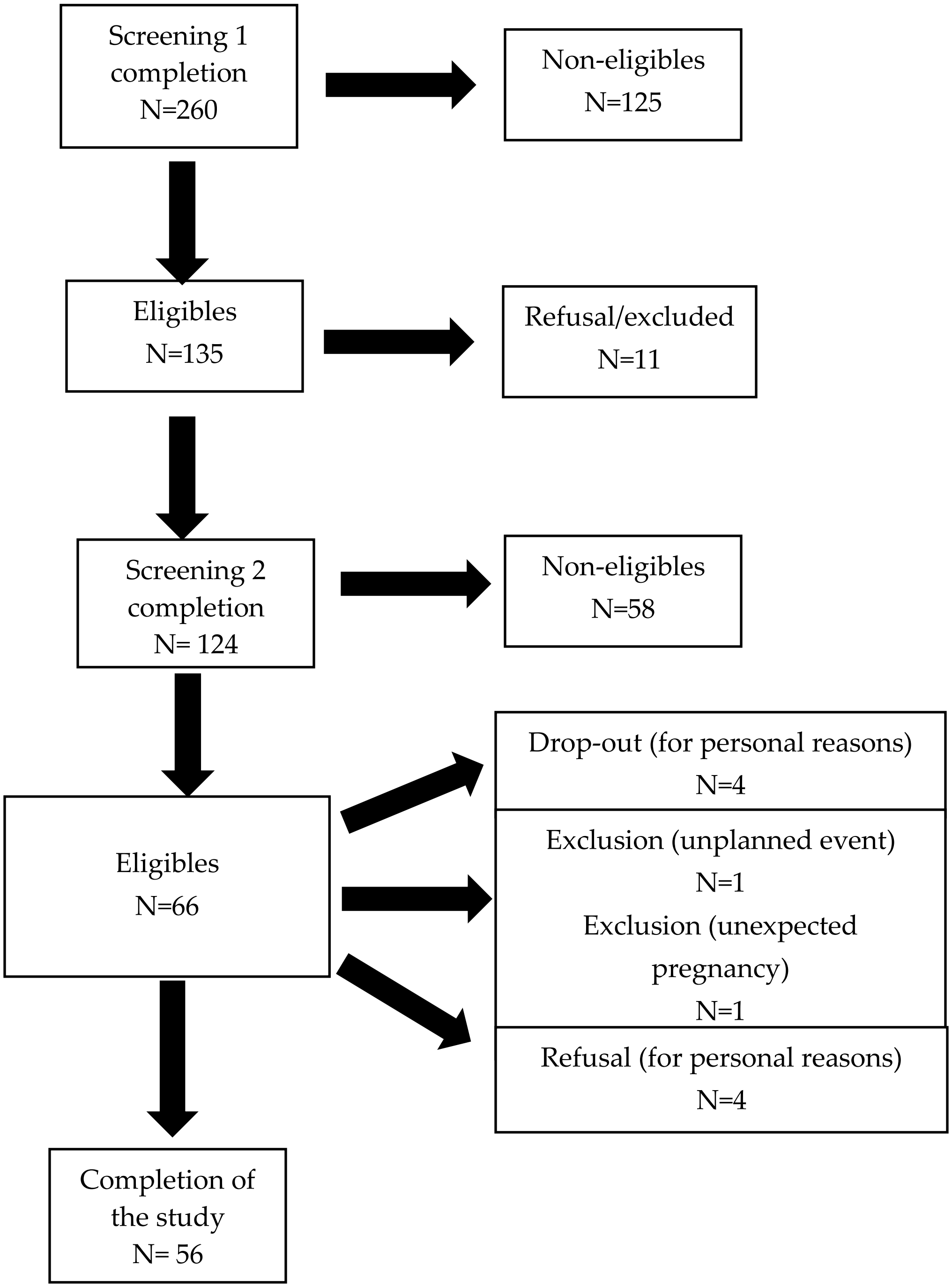


Supplementary Figure S1. Flow chart.
